# New live attenuated tuberculosis vaccine MTBVAC induces trained immunity and confers protection against experimental lethal pneumonia

**DOI:** 10.1371/journal.ppat.1008404

**Published:** 2020-04-02

**Authors:** Raquel Tarancón, Jorge Domínguez-Andrés, Santiago Uranga, Anaísa V. Ferreira, Laszlo A. Groh, Mirian Domenech, Fernando González-Camacho, Niels P. Riksen, Nacho Aguilo, José Yuste, Carlos Martín, Mihai G. Netea

**Affiliations:** 1 Department of Microbiology, Faculty of Medicine, University of Zaragoza, Zaragoza, Spain; 2 CIBERES and Research Network on Respiratory Diseases, Spanish Ministry of Health and Instituto de Salud Carlos III, Madrid, Spain; 3 Department of Internal Medicine and Radboud Center for Infectious diseases (RCI), Radboud University Nijmegen Medical Centre, Geert Grooteplein 8, Nijmegen, the Netherlands; 4 Instituto de Ciências Biomédicas Abel Salazar (ICBAS), Universidade do Porto, Porto, Portugal; 5 Centro Nacional de Microbiología, Instituto de Salud Carlos III, Madrid, Spain; 6 Servicio de Microbiología, Hospital Miguel Servet, ISS Aragón, Zaragoza, Spain; 7 Department for Genomics & Immunoregulation, Life and Medical Sciences Institute (LIMES), University of Bonn, Bonn, Germany; 8 Human Genomics Laboratory, Craiova University of Medicine and Pharmacy, Craiova, Romania; McGill UniversityHealth Centre, CANADA

## Abstract

Among infectious diseases, tuberculosis is the leading cause of death worldwide, and represents a serious threat, especially in developing countries. The protective effects of *Bacillus Calmette-Guerin* (BCG), the current vaccine against tuberculosis, have been related not only to specific induction of T-cell immunity, but also with the long-term epigenetic and metabolic reprogramming of the cells from the innate immune system through a process termed trained immunity. Here we show that MTBVAC, a live attenuated strain of *Mycobacterium tuberculosis*, safe and immunogenic against tuberculosis antigens in adults and newborns, is also able to generate trained immunity through the induction of glycolysis and glutaminolysis and the accumulation of histone methylation marks at the promoters of proinflammatory genes, facilitating an enhanced response after secondary challenge with non-related bacterial stimuli. Importantly, these findings in human primary myeloid cells are complemented by a strong MTBVAC-induced heterologous protection against a lethal challenge with *Streptococcus pneumoniae* in an experimental murine model of pneumonia.

## Author summary

*Mycobacterium tuberculosis* has been causing infections in our species and our ancestors for at least thousands of years. Still today, the numbers of people affected by tuberculosis are alarming with more than 1,4 million deaths per year, representing the first cause of death by infectious disease worldwide. Despite immense research efforts, Bacille Calmette-Guerin (BCG), a vaccine based on a live attenuated form of *Mycobacterium bovis* that was developed one century ago, remains the only licensed vaccine against tuberculosis. Several independent works have shown that BCG induces protection not only against tuberculosis, but also against other infections through the induction of epigenetic and metabolic effects in the cells of the innate immune system, what has been termed trained immunity. Here we describe how MTBVAC, a new vaccine against tuberculosis based on a genetically modified form of the human pathogen *Mycobacterium tuberculosis*, that has shown similar safety profiles and improved protection than BCG in preclinical studies, is able to induce trained immunity in human cells and grant protection against a heterologous model of pneumococcal infection in mice. These results underline the potential of MTBVAC as a candidate for universal vaccination against tuberculosis or treatment against bladder cancer, representing a potential alternative to the current BCG vaccines.

## Introduction

For thousands of years *Mycobacterium tuberculosis* (Mtb) has caused considerable infectious burden for our species [1,2]. Even nowadays, tuberculosis (TB) remains the first cause of death by infectious disease killing more than 1,4 million people [3]. According to the last report from the WHO, 23% of the global population presents latent TB infection (LTBI), meaning that they are infected by the pathogen but they have not yet become ill and cannot transmit the infection [3,4]. There are around 10 million new cases every year, of which almost half a million are multidrug-resistant TB, characterized by resistance to isoniazid and rifampicin, the two main pharmacological treatments against the disease. In this scenario, it is fundamental to investigate and develop new therapeutic approaches against TB. Despite immense research efforts into new vaccines, BCG is still the only licensed vaccine against TB (*Mycobacterium bovis* Bacillus Calmette-Guerin; BCG), despite the fact that it was approved for its use in humans almost a century ago [5]. BCG is administered worldwide as a single dose of intradermal inoculation, providing protection against disseminated forms of TB such as milliary TB in infants, but offering variable degrees of protection against pulmonary and LTBI in adults of both sexes and all ages ranging from 0% to 80% depending on the setting [6].

Following decades of intensive research, a number of vaccines against TB and their corresponding adjuvants have been tested, though to date none of them have been found to be more effective than BCG [7]. The greatest challenge in the development of vaccines against TB is to understand the mechanisms by which Mtb evades and escapes the immune responses of the host. MTBVAC is the first live attenuated vaccine, genetically modified, based on the human pathogen Mtb. MTBVAC meets the Geneva consensus requirements to enter into clinical trials in humans, showing similar safety and bio-distribution profiles as BCG [8]. In preclinical studies, MTBVAC confers higher protection against TB than BCG [9,10]. Apart from the specific protection against Mtb infections, BCG also mediates a series of non-specific immunomodulatory effects in monocytes by means of metabolic and epigenetic reprogramming of innate immune cells through a process termed "trained immunity" [11,12]. This process has been related to a decreased incidence of neonatal sepsis and respiratory infections in children vaccinated with BCG [13], as well as the therapeutic efficacy of vesical BCG instillations in patients with bladder cancer [14].

Considering the substantial additional benefits of an up to 50% decrease in mortality due to the non-specific effects of BCG in children [15–17], it is crucial to establish whether any new TB vaccines currently under investigation are also able to induce trained immunity and initiate similar heterologous protective effects. It is not known whether MTBVAC can induce trained immunity and protect against non-specific infections, although positive effects against bladder cancer have been described in preclinical studies [18]. In the present study, we assessed for the first time the potential of MTBVAC to induce trained immunity and protect against a heterologous model of pneumococcal pneumonia in mice.

## Results

### MTBVAC exerts comparable immunomodulatory effects to BCG in human monocytes and PBMCs

**Fig 1:**
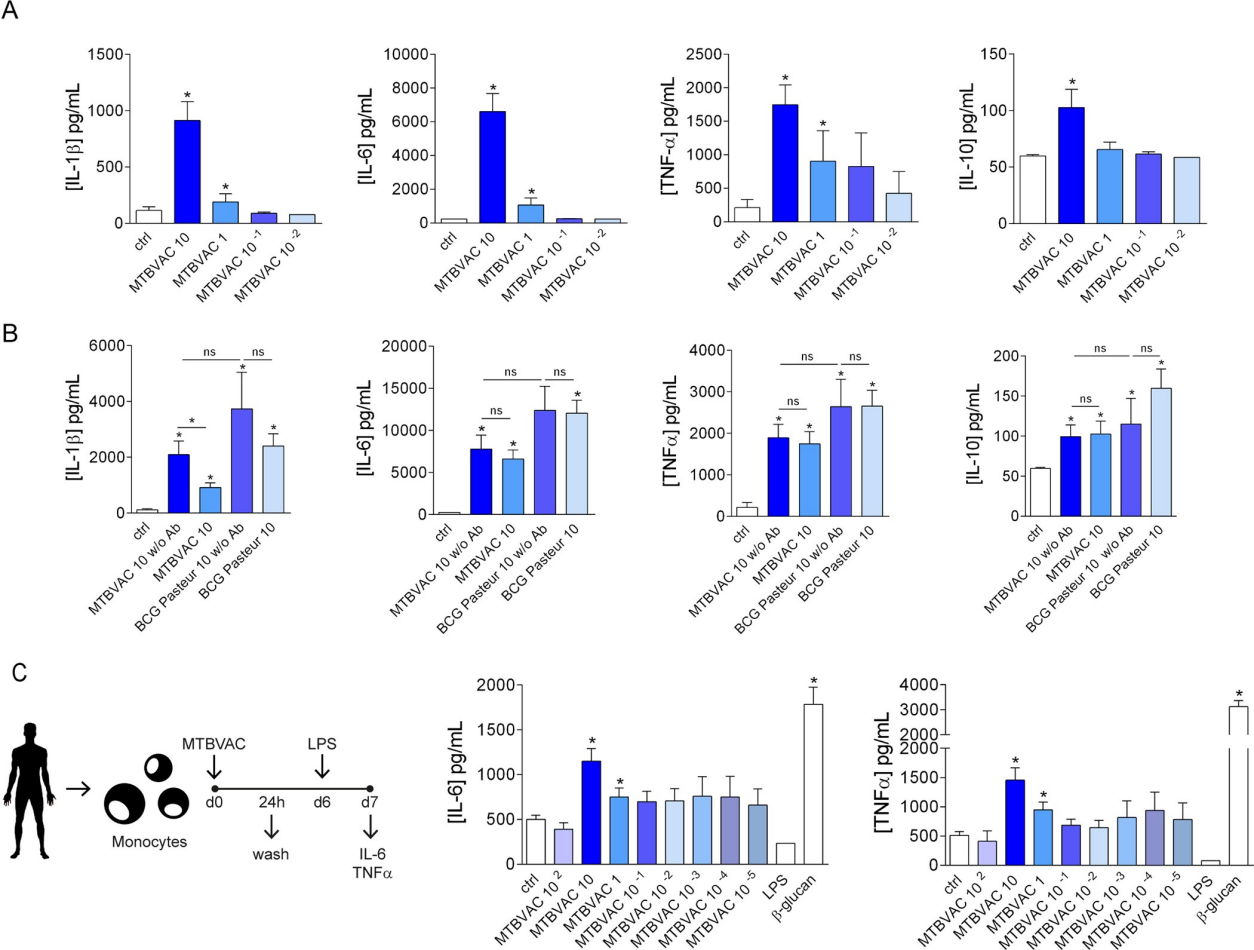
MTBVAC stimulation enhances cytokine production and trained immunity in human monocytes. (A) IL-1β, IL-6, TNFα and IL-10 production by human monocytes 24 h after stimulation with different concentrations of MTBVAC. (B) IL-1β, IL-6, TNFα and IL-10 production by human monocytes 24 h after stimulation with MTBVAC or BCG Pasteur, with or without antibiotic in the medium. (C) IL-6 and TNFα produced by human monocytes stimulated with MTBVAC or BCG Pasteur for 24 h and restimulated with LPS 6 days later following the depicted scheme. Mean ± SEM, n = 6–9 (from 6 different individual donors); pooled from 2–3 independent experiments with 3 individual donors each. *p<0.05, Wilcoxon signed-rank test, compared to the control group unless otherwise stated. Concentration numbers refer to MOI (multiplicity of infection; ratio MTBVAC or BCG Pasteur per monocyte). w/o Ab: without antibiotic (gentamicin); ns: not significant; ctrl: control group.

First, we assessed the potential of MTBVAC to stimulate human cells *in vitro*. In a setting of acute stimulation of human monocytes, we found that MTBVAC had a dose-dependent effect on the stimulation of the pro-inflammatory cytokines IL-1β, IL-6 and TNFα by human monocytes (Fig 1A). Stimulation of the cells with a high dose of MTBVAC also induced the production of low amounts of IL-10. Since MTBVAC is a live attenuated strain of Mtb, we also assessed if the presence or absence of the antibiotic gentamicin in the culture medium, which may affect the viability of the mycobacteria, had any influence in the induction of cytokine production. We found that the withdrawal of this antibiotic from the medium did not exert any remarkable influence in cytokine production after acute stimulation, with the exception of a decrease in the concentrations of IL-1β, most likely attributable to the presence of extracellular mycobacteria in the absence of antibiotic (Fig 1B and S1A Fig). We compared these effects with BCG Pasteur [19]. We found that MTBVAC induced the production of IL-1β, IL-6, TNFα and IL-10 comparable to the BCG strain (Fig 1B and S1B Fig). In the case of peripheral blood mononuclear cells (PBMCs), stimulation with MTBVAC led not only to the production of the aforementioned cytokines, but also to that of IFNγ, IL-17 and IL-22, 7 days after the challenge (S2 Fig).

In order to assess the potential of MTBVAC in the induction of innate immune memory, we followed a well-described protocol for the induction of trained immunity *in vitro* [20] and stimulated human monocytes with different doses of MTBVAC for 24h, after which we washed the stimulus and let the cells rest for 5 days. On day 6 we restimulated the cells with lipopolysaccharide (LPS) from *Escherichia coli* for 24h. In agreement with previous reports [12,20,21], we evaluated the induction of trained immunity by measuring the concentrations of IL-6 and TNFα in the supernatants of these cells. We observed a clear dose-dependent effect of MTBVAC in the induction of trained immunity in human monocytes, with a 10:1 MTBVAC:monocyte ratio being the most effective (Fig 1C). Similarly to what was observed after acute stimulation, the induction of trained immunity by MTBVAC was not affected by the presence of gentamicin in the medium (Fig 1C and S3 Fig). The amounts of IL-6 and TNFα produced in monocytes trained with MTBVAC were comparable to those seen after training with BCG Pasteur after secondary challenge with LPS (S3 Fig). In the absence of gentamicin, we observed a decrease in cytokine production after stimulation with BCG Pasteur, which can be explained by the toxic effects caused by the survival and proliferation of a high dose mycobacteria in the absence of antibiotic after one week of culture (S3 Fig). Overall, these results confirmed that stimulation with MTBVAC triggers the induction of trained immunity in human cells.

### MTBVAC induces metabolic reprogramming in human monocytes

**Fig 2:**
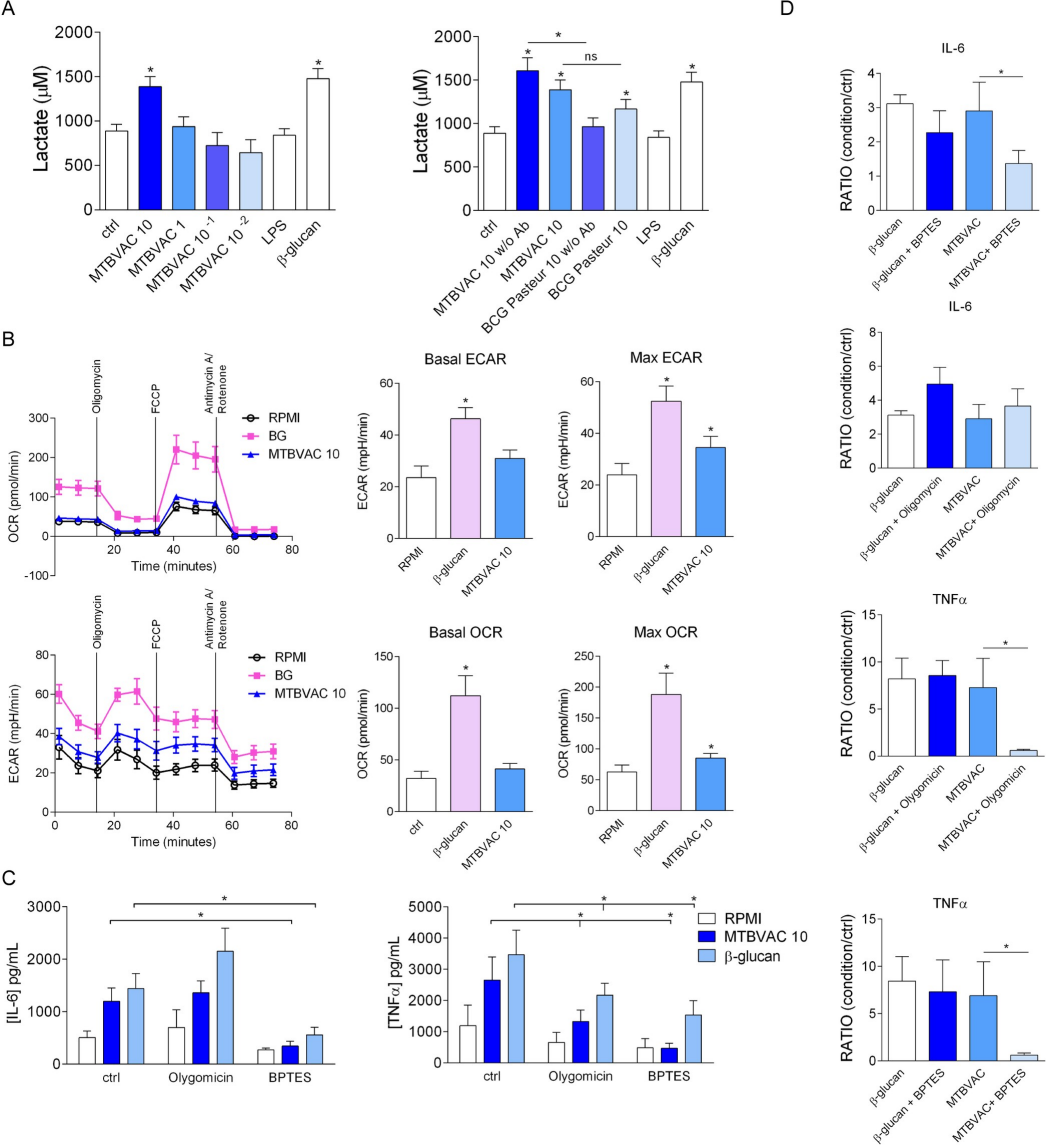
MTBVAC induces trained immunity through modulation of glycolysis and glutaminolysis. (A) Lactate production by human monocytes 6 days after 24h-stimulation with different concentrations of MTBVAC or BCG Pasteur with or without antibiotic in the medium (n = 6–9; pooled from 2–3 independent experiments). (B) Basal and maximum (Max) oxygen consumption rate (OCR) and extracellular acidification rate (ECAR) of monocytes were determined 6 days after 24h-stimulation with β-glucan or MTBVAC by extracellular flux measurements (mean ± SEM, n = 6; pooled from 2 independent experiments). *p<0.05, Wilcoxon signed-rank test, compared to the control group unless otherwise stated. (C) IL-6 and TNFα produced by human monocytes pretreated with oligomycin or BPTES and subsequently trained with MTBVAC, or β-glucan for 24 h and restimulated with LPS 6 days later. Mean ± SEM, n = 6; pooled from 2 independent experiments with 3 individual donors each. *p<0.05, Wilcoxon signed-rank test, compared to the control group. BG: β-glucan; FCCP: Carbonyl cyanide 4-(trifluoromethoxy) phenylhydrazone. w/o Ab: without antibiotic (gentamicin); ctrl: control group.

The induction of trained immunity is linked with the reprogramming of the metabolic landscape of the cells [22]. In accordance with previous reports describing that BCG-mediated trained immunity upregulates glycolytic pathways in human monocytes, we found that MTBVAC increased the production of lactate by these cells to levels comparable with those induced by stimulation with β-glucan, the gold standard for *in vitro* trained immunity induction (Fig 2A and S4A Fig). In addition, using the Seahorse technology to assess the bioenergetic profiles of the cells [23], we observed a moderate increase in the oxygen consumption rate (OCR), and extracellular acidification rate (ECAR), confirming an enhanced metabolic activity in the human monocytes exposed to MTBVAC (Fig 2B). Previous research has shown that trained immunity in monocytes relies on the induction of glutaminolysis and oxidative phosphorylation [24]. Glutaminolysis consists of the conversion of the amino acid glutamine into α-ketoglutarate, which gets in the mitochondria and enters TCA cycle, fueling oxidative phosphorylation and increasing ATP formation [12,25]. We confirmed the relevance of these metabolic pathways in the induction of trained immunity by MTBVAC by performing experiments with pharmacological inhibitors of specific metabolic pathways. Inhibition of the activity of the TCA cycle with oligomycin, an inhibitor of oxidative phosphorylation, and inhibition of glutaminolysis with BPTES, a glutaminase inhibitor, impeded the production of IL-6 and TNFα after secondary stimulation of monocytes with LPS (Fig 2C), highlighting the importance of these metabolic pathways in the induction of trained immunity with MTVBAC. The comparison of the changes in the OCR and ECAR induced by BCG and MTBVAC showed that the metabolic rewiring induced by these two different stimuli is similar (S4B Fig). Of note, the stimulation of monocytes with MTBVAC and BCG Pasteur induced the production of reactive oxygen species (ROS) by monocytes (S5 Fig), similarly to what was described for other BCG strains [20]. Incubation of the cells with 6-AN, an inhibitor of the pentose phosphate pathway, a metabolic pathway parallel to glycolysis which generates NADPH, pentoses and ribose 5-phosphate, a precursor for the synthesis of nucleotides, did not induce significant changes in ROS production in comparison to untreated cells, so this metabolic pathway was not involved in the generation of ROS in response to MTBVAC or BCG Pasteur.

### MTBVAC induces epigenetic reprogramming in human monocytes

**Fig 3:**
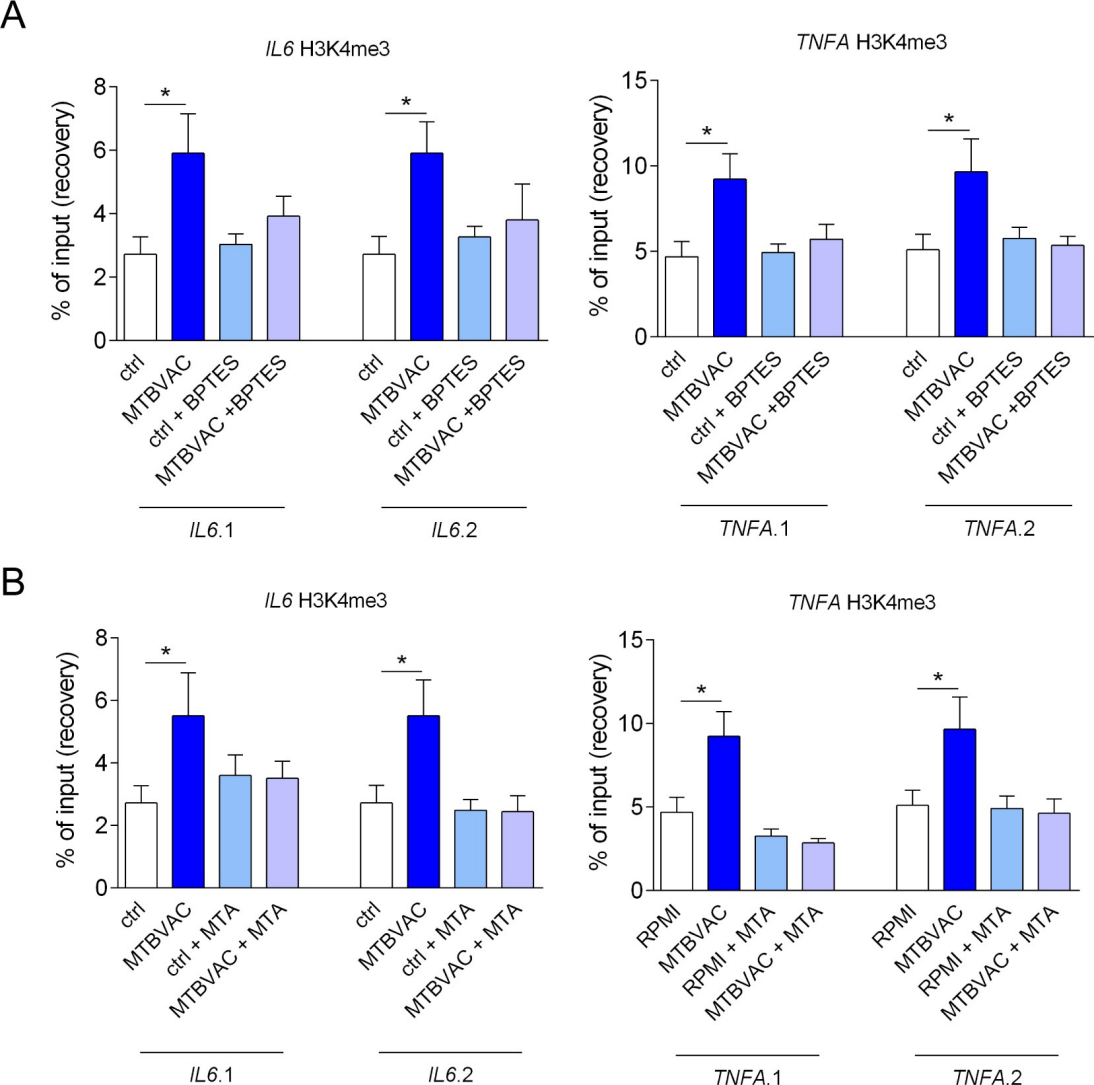
MTBVAC enhances accumulation of H3K4me3 at the promoters of IL-6 and TNFα. (A-B) After 6 days of culture, H3K4me3 marks were assessed at the level of promoters of *TNFΑ* and *IL6* with two different pairs of primers each. Cells were pre-treated with BPTES (A) or MTA (B). Mean ± SEM, n = 6; pooled from 2 independent experiments with 3 individual donors each. *p<0.05, Wilcoxon signed-rank test, compared to the control group; ctrl: control group.

Enrichment in histone methylation marks in the promoter regions of pro-inflammatory genes, such as *IL6* and *TNFΑ*, associated with open chromatin and enhanced transcription is a hallmark of innate immune memory, and has been widely used to test the epigenetic effects of different stimuli to induce trained immunity [12,26,27]. Consequently, we analyzed the deposition of histone 3 lysine 4 trimethylation (H3K4me3) marks on the promoter regions of *IL6* and *TNFΑ* genes after stimulation of the cells with MTBVAC. The results of these experiments confirmed that training with MTBVAC led to the accumulation of H3K4me3 marks at the promoter regions of *IL6* and *TNFΑ* to an extent comparable to that induced by BCG and β-glucan (S6 Fig). In order to assess the relevance of the interplay between metabolism and epigenetic reprogramming, we analyzed how the inhibition of glutamine breakdown with BPTES affected the accumulation of H3K4me3 in the genes studied. We observed that inhibition of glutaminolysis dampened the accumulation of H3K4me3 in the promoter regions of *IL6* and *TNFΑ* (Fig 3A). The effects observed with BPTES were comparable to those seen after treatment with MTA, a methyltransferase inhibitor, reported to block the deposition of H3K4me3 (Fig 3B), confirming the relevance of the interplay between metabolic and epigenetic reprogramming in the induction of trained immunity by MTBVAC.

### MTBVAC induces trained immunity *in vivo*

**Fig 4:**
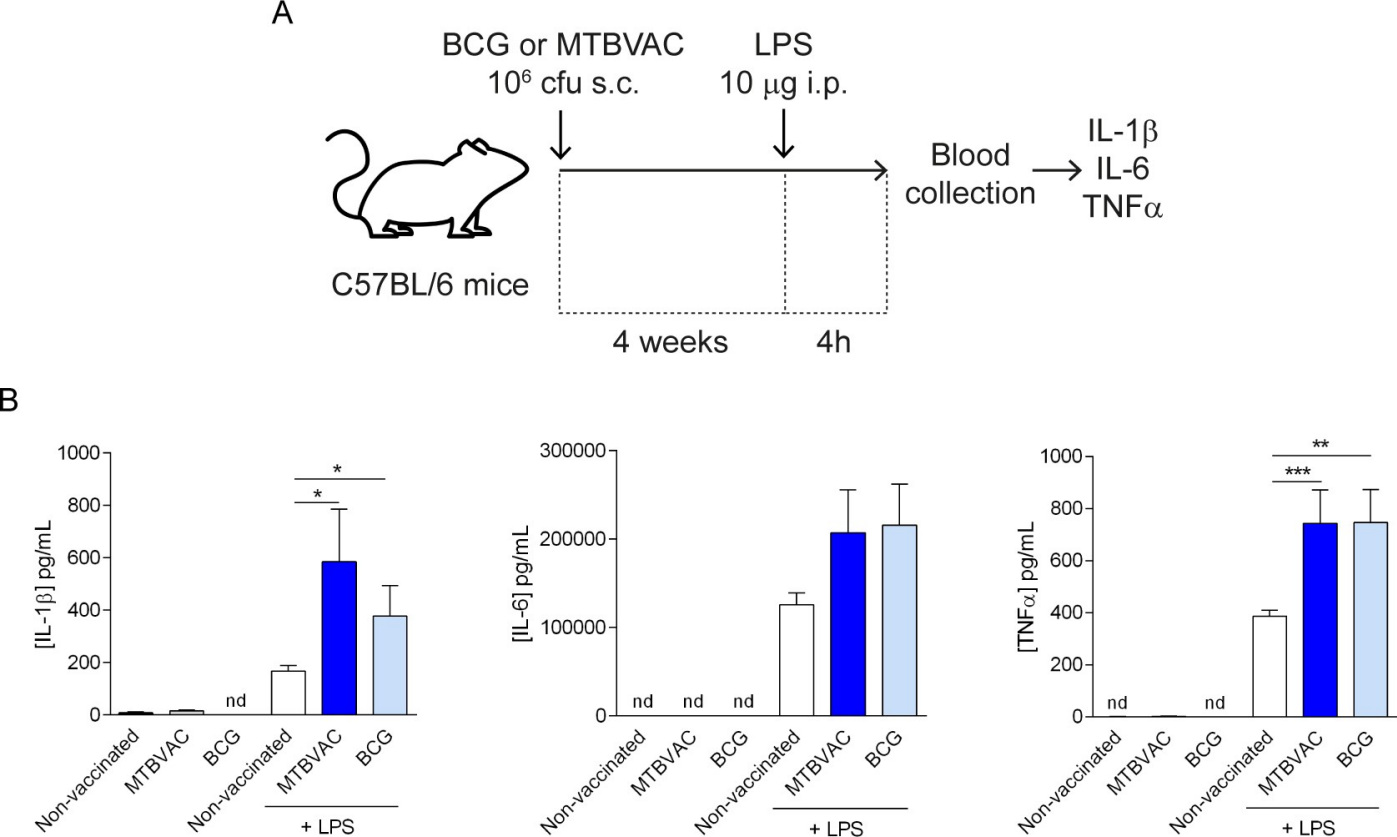
Vaccination with MTBVAC enhances cytokine production after secondary heterologous stimulation. (A) Protocol of vaccination with BCG or MTBVAC and restimulation with LPS. (B) IL-1β, IL-6 and TNFα in circulating serum from mice subject to the protocol described in (A). Mean ± SEM, n = 7 (from 7 different mice). *p<0.05, Mann-Whitney test, compared to the control group (non-vaccinated). nd: non-detectable.

**Fig 5:**
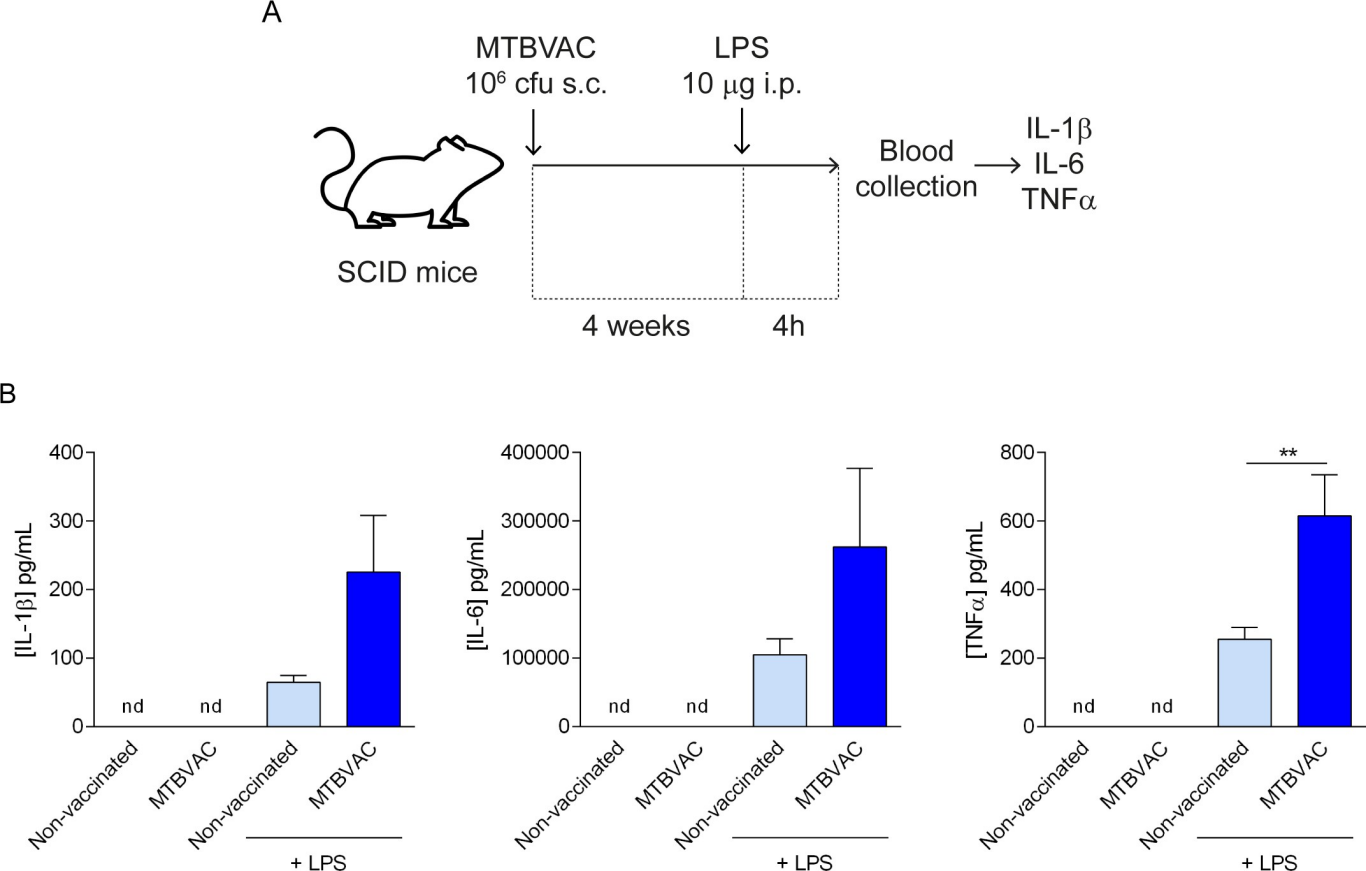
Vaccination with MTBVAC induces enhanced cytokine production after secondary heterologous stimulation in SCID mice. (A) Protocol of vaccination with MTBVAC and restimulation with LPS in SCID mice. (B) IL-1β, IL-6 and TNFα in circulating serum from mice subject to the protocol described in (A). Mean ± SEM, n = 6 (from 6 different mice). **p<0.01, Mann-Whitney test. nd: non-detectable.

In order to confirm the relevance of these mechanisms *in vivo*, a group of C57BL/6 mice were vaccinated with a clinically relevant dose of MTBVAC [8,28,29] or BCG subcutaneously. After 4 weeks, these mice were challenged with LPS, and the levels of their proinflammatory cytokines were measured in serum (Fig 4A). We found that mice vaccinated only with MTBVAC or BCG did not present measurable levels of IL-1β, IL-6 or TNFα, 4 weeks after the inoculation, whereas animals that were vaccinated with MTBVAC or BCG, and subsequently challenged with LPS presented higher levels of circulating IL-1β, IL-6 and TNFα than mice that had not been vaccinated prior to the secondary challenge (Fig 4B). A similar experiment conducted in SCID mice, which do not have functional T and B cells [30], showed that SCID mice vaccinated with MTBVAC presented higher production of IL-1β, IL-6 and TNFα after heterologous challenge with LPS, confirming that the enhanced responsiveness seen in mice vaccinated with MTBVAC after secondary stimulation relies on the activation of the innate immune system (Fig 5). Overall, our results confirm that MTBVAC presents comparable trained immunity-inducting capacities than a BCG strain employed in human vaccination.

### MTBVAC confers unspecific protection against lethal pneumonia

**Fig 6:**
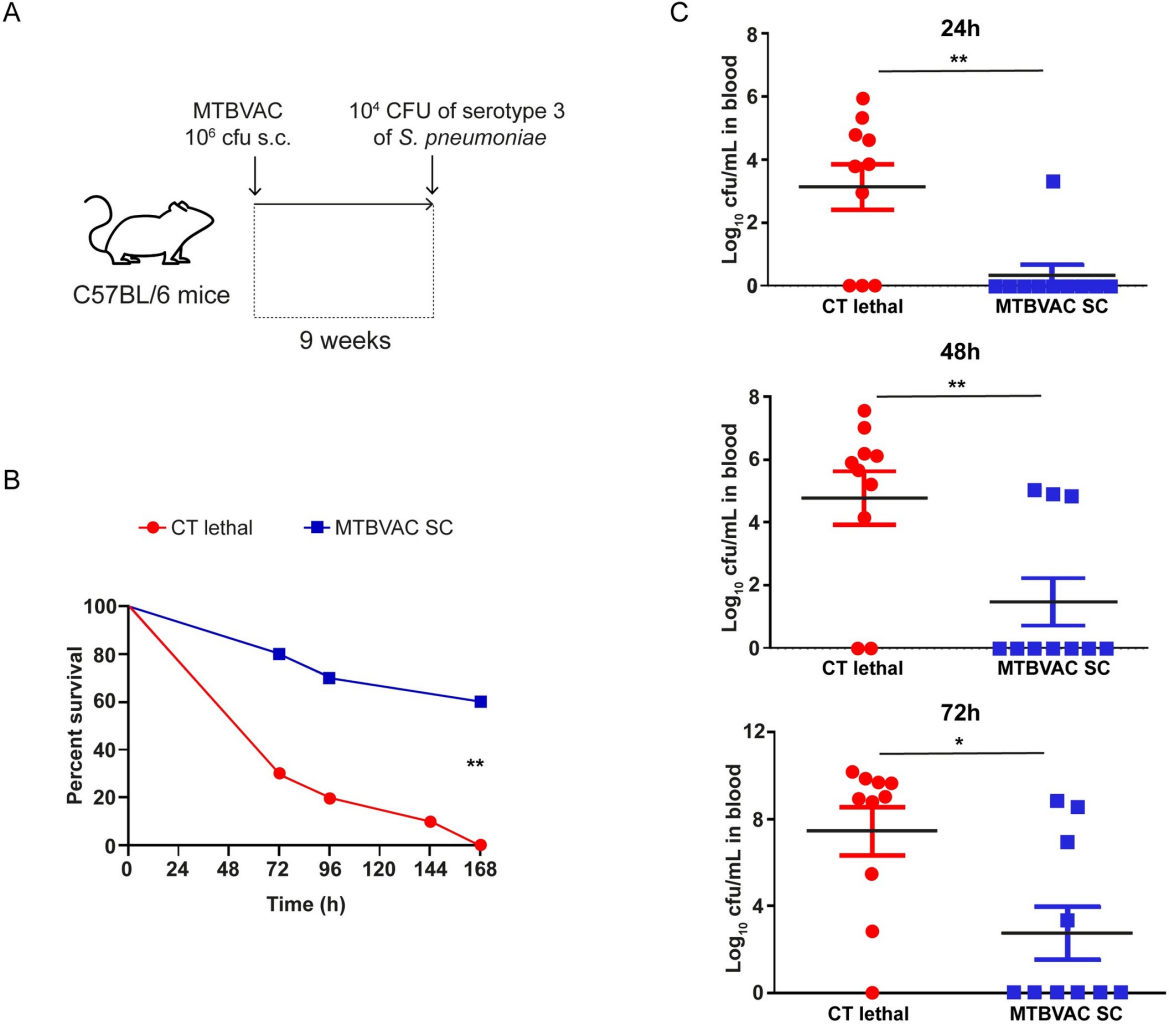
Vaccination with MTBVAC protects against pneumococcal pneumonia. (A) Protocol of vaccination with BCG or MTBVAC and subsequent infection with *S*. *pneumoniae*. (B) Survival of mice subcutaneously (SC) vaccinated with 10^6^ CFU of MTBVAC (blue squares) or placebo as lethal control (red circles) and infected by the intranasal route with 1×10^4^ CFU of serotype 3 of *S*. *pneumoniae*. (C) Bacterial levels in blood at 24 h, 48 h and 72 h of the lethal control and MTBVAC vaccinated group. **p<0.01, Log-rank (Mantel-Cox test) for the survival experiment. *p<0.05 or **p<0.01 by two-tailed Student’s t test; CT lethal: control lethal.

Trained immunity has been suggested to be responsible for the non-specific protection against heterologous pathogens conferred by some vaccines, including BCG. To assess whether this is the case also for MTBVAC, we administered either vaccine or saline control to C57BL/6 mice, and 9 weeks later we delivered intranasally a lethal challenge of *Streptococcus pneumoniae* (Fig 6A). Our results showed that 60% of the vaccinated mice survived at the end of the experiment, whereas in the non-vaccinated group all the animals died. Analysis of bacterial levels in blood indicated absence of live bacteria in the surviving animals (Fig 6B), suggesting that MTBVAC-induced sterilizing immunity against *S*. *pneumoniae*. As a result of MTBVAC vaccination, pneumococcal pneumonia was efficiently controlled, abrogating bacterial replication and dissemination in the host, contributing to reduce the severity and mortality of the invasive disease process.

## Discussion

There is an urgent need to increase and improve the repertoire of effective vaccines against TB. Even though there are a number of TB vaccines currently being tested in clinical trials, the only licensed TB vaccine in use today is BCG, an attenuated form of *M*. *bovis* which has been in use since the early 20^th^ century. BCG protects against disseminated forms of TB, but it is much less effective against pulmonary TB, the form responsible for the transmission and spreading of the disease. Therefore, it is crucial to find new vaccination strategies that afford protection against this form of TB. In this regard MTBVAC, a live attenuated vaccine based on *M*. *tuberculosis*, is a reliable candidate for vaccination, with similar safety and bio-distribution profiles as BCG, but containing a much larger repertoire of epitopes recognized by human cells, including several antigens that are missing in BCG [8,9].

However, before a new prime vaccine could replace BCG in public health programs, an important issue needs to be addressed. A growing body of scientific literature has proven that BCG vaccination is able to induce a series of heterologous protective effects against other non-TB related infections, especially neonatal sepsis and respiratory tract infections [13]. These non-specific effects of BCG vaccination rely on the induction of trained immunity, a process through which the cells of the innate immune system undergo metabolic and epigenetic reprogramming that allow for an increased responsiveness against immunogenic stimuli [31]. In this study we show that the live attenuated vaccine MTBVAC is able to activate pro-inflammatory cytokines and trigger long-term reprogramming of innate immune cells at levels comparable to BCG. Human monocytes stimulated with MTBVAC underwent metabolic and epigenetic changes that led to an enhanced secondary response upon restimulation with LPS. Stimulation of cells with MTBVAC induced an increase in the glycolytic activity of the cells, similarly to what has been previously described for BCG and for other stimuli that induce trained immunity, such as β-glucan [32] or oxLDL [33]. This increase in glycolysis was accompanied by an enhanced oxidative phosphorylation TCA cycle activity, as measured by OCR. Of note, we observe that BCG Pasteur tends to induce more acute cytokine production in the presence of gentamicin than in the absence of it. This can be explained by the fact that BCG Pasteur is the most reactogenic strain and it can exert cellular toxicity in high concentrations. With a high dose of BCG Pasteur (BCG:monocyte ratio of 10:1) in the absence of antibiotic, mycobacteria can present enhanced survival and induce toxicity to the monocytes after several days of culture. In addition, extracellular bacteria are able to survival extracellularly in the absence of gentamicin, while in the presence of this antibiotic in the medium, only the effect of intracellular bacteria is observed. Therefore, we speculate that a high dose of BCG in the absence of gentamicin may be toxic for the monocytes after several days of culture, which could explain the lower cytokine and lactate production seen after following the usual protocols for the induction of trained immunity in these cases.

Arts. et al described that the induction of trained immunity by BCG in monocytes relies on glutaminolysis, leading to accumulation of the TCA cycle metabolite fumarate, that in turn inhibits the H3K4 demethylase KDM5 [12]. We validate here these findings by showing that the induction of trained immunity by MTBVAC relies on the induction of glutaminolysis, and that the inhibition of this pathway impairs trained immunity in human monocytes. The second pillar of the induction of trained immunity is the deposition of epigenetic marks on the promoter regions of pro-inflammatory genes involved in the induction of innate immune responses [34]. In line with this, treatment with MTBVAC led to a greatly increased presence of H3K4me3, a histone modification related with an enhanced and more robust gene transcription after stimulation [35]. The increased presence of this epigenetic mark in the induction of innate responses [34] is in accordance with the increased responsiveness of MTBVAC-stimulated monocytes observed after secondary stimulation. Importantly, a fully functional glutaminolysis pathway is fundamental for the induction of epigenetic reprogramming of monocytes by MTBVAC, since the impairment of this pathway led to a loss of H3K4me3 accumulation in the promoter regions of *IL6* and *TNFΑ*.

The interplay between cellular metabolism and epigenetic mechanisms within immune cells are gaining attention in recent years and has been related not only with the induction of a protective immune response against infectious diseases and cancer [31,36], but also with the development of atherosclerosis, diabetes or other diseases related with excessive inflammation [37,38]. The *in vivo* functional relevance of these mechanisms was demonstrated in mice, where a single dose of MTBVAC vaccination resulted in an enhanced proinflammatory response against heterologous stimulation 4 weeks after vaccination. This effect is likely to happen through metabolic and epigenetic reprogramming of the hematopoietic niche, as it has been recently described for the induction of trained immunity with β-glucan [39] and also with BCG vaccination [40].

The potential clinical relevance of our data is demonstrated by the observation that subcutaneous MTBVAC vaccination strongly protects against a lethal experimental infection with *S*. *pneumoniae*. The development of invasive pneumococcal disease (IPD) is attributable to many factors including virulence components of the bacterium and the efficacy of the host immune response to clear the infection process. Failures in the detection and control of *S*. *pneumoniae* in the lower respiratory tract or the systemic circulation lead to severe pneumonia or disseminated infection which are associated to increased mortality rates [41]. In this sense, trained immunity driven by immunization with MTBVAC triggered the immune response against a subsequent infection by *S*. *pneumoniae* controlling the propagation from the lung to the bloodstream and protecting against the systemic infection.

More studies are required to elucidate the complexity of the immune mechanisms behind this protective effect, but the more robust cytokine production induced by MTBVAC vaccination, that is crucial for the subsequent activation of host defense, is likely to have played a major role. Indeed, mice deficient in CCL2, the main monocyte-recruiting cytokine, are highly sensitive to *S*. *pneumoniae* infection [42]. Noteworthy, the *S*. *pneumoniae* clinical isolate used in the present study was a serotype 3 strain which is currently one of the most prevalent serotypes causing IPD and pneumonia worldwide[43,44] and to which current PCV13 conjugate vaccine efficacy against this serotype is limited [45].

Collectively, the evidence presented in this manuscript underline that MTBVAC is able to exert metabolic and epigenetic immunomodulatory effects through mechanisms similar to those of BCG. In line with this, vaccination with MTBVAC is likely to recapitulate the non-specific protective effects of BCG vaccination against heterologous infections, while at the same time improving the specific responses against Mtb infection. All together, these results underline the potential of MTBVAC as a reliable candidate for mass vaccination, representing a possible alternative to the currently available BCG vaccines if a better protection against TB disease could be demonstrated in Phase 3 efficacy trials.

## Materials and methods

### Peripheral blood mononuclear cell and monocyte isolation

PBMC isolation was performed by differential density centrifugation over Ficoll-Paque (GE Healthcare). Percoll isolation of monocytes was performed as previously described [46]. Briefly, 150–200 x 10^6^ PBMCs were layered on top of a hyper-osmotic Percoll solution and centrifuged for 15 min at 580g. The interphase layer was isolated and cells were washed with cold PBS. Cells were re-suspended in RPMI medium Dutch modified (Invitrogen) supplemented with 50 μg/mL gentamicin, 2 mM Glutamax, and 1 mM pyruvate, and counted. An extra purification step was added by adhering Percoll-isolated monocytes to polystyrene flat bottom plates (Corning) for 1 h at 37˚C; a washing step with warm PBS was then performed to yield maximal purity.

### Cytokine stimulation and Trained Immunity experiments

5 x 10^5^ PBMCs/mL or 10^5^ monocytes were added to flat-bottom 96-well plates (Greiner). Cells were stimulated with RPMI, β-glucan (1 μg/ml), LPS (10 ng/mL) or different multiplicity of infection (MOI) of BCG Pasteur (strain 1173P2, Institut Pasteur Paris, France [19]) and MTBVAC [28] (University of Zaragoza) for 24h-incubation at 37°C with or without antibiotic, as indicated. Thereafter, the supernatant was discarded, cells were washed and the medium replaced with fresh RPMI with 10% human serum. After 6 days at 37°C, the supernatant was discarded and the cells were stimulated with *E*. *coli* LPS (10 ng/mL) for an additional 24 h. Subsequently, the supernatants were stored at −20°C until ELISA was performed.

The mycobacterial strains used were growth until logarithmic phase, at 37°C in Middlebrook 7H9 broth (Difco) supplemented with ADC 10% (0.2% dextrose, 0.5% BSA fraction V, 0.0003% beef catalase) (Difco) and 0.05% (v/v) Tween-80 (Sigma), or on solid Middlebrook 7H11 (Difco) supplemented with ADC 10%. Then, pellet was collected and resuspended in 0.1 volume of PBS-Tween 80 at 0.05% respect to the initial culture volume. Aggregates were eliminated by centrifugation at 1400 rpm during 5min, supernatant collected and a final concentration of 5% of glycerol was added. Aliquots of 0.5 ml were stored at -80°C, subsequent plating was performed in order to count the CFUs contained in the preparation.

### Cytokine measurements

Cytokine production from human cells was determined in supernatants using commercial ELISA kits for IL-1β, IL-6, TNFα, IL-10, IFNγ, IL-17 and IL-22 (R&D Systems, Minneapolis, MN) following the instructions of the manufacturer.

### Chromatin immunoprecipitation

Purified cells were fixed with 1% formaldehyde (Sigma) at a concentration of approximately 10^6^ cells/ml. Fixed cell preparations were sonicated using a Diagenode Bioruptor UCD-300 for 3x10 min (30 s on; 30 s off). 67 μl of chromatin (1 million cells) were incubated with 229 μl dilution buffer, 3 μl protease inhibitor cocktail and 0.5–1 μg of H3K4me3 antibody (Diagenode) and incubated overnight at 4°C with rotation. Protein A/G magnetic beads were washed in dilution buffer with 0.15% SDS and 0.1% BSA, added to the chromatin/antibody mix and rotated for 60 min at 4°C. Beads were washed with 400 μl buffer for 5 min at 4C with five rounds of washes. After washing chromatin was eluted using elution buffer for 20 min. Supernatant was collected, 8 ml 5M NaCl, 3 ml proteinase K were added and samples were incubated for 4 h at 65°C. Finally, samples were purified using QIAGEN; Qiaquick MinElute PCR purification Kit and eluted in 20 ml EB.

### ROS production

Oxygen radical production levels were evaluated using luminol-enhanced chemiluminescence and determined in a luminometer. Monocytes trained with RPMI, MTBVAC or BCG Pasteur were stimulated with RPMI or opsonized zymosan (1 mg/ml). Luminol was added to each well in order to start the chemiluminescence reaction. Each measurement was carried out in at least duplicate repetitions. Chemiluminescence was determined every 145 s at 37˚C for 1 h. Luminescence was expressed as relative light units (RLU) per second.

### Metabolic measurements

1 × 10^7^ monocytes were trained in 10 cm Petri dishes (Greiner) in 10 mL of RPMI medium with or without stimuli for 24 h, washed with warm PBS and incubated in RPMI culture medium at 37°C, 5% CO_2_. Following 5 days in culture, cells were detached with Versene solution (ThermoFisher Scientific) and 1 x 10^5^ cells were plated to overnight-calibrated cartridges in assay medium (DMEM with 0.6 mM glutamine, 5 mM glucose and 1 mM pyruvate [pH adjusted to 7.4]) and incubated for 1h in a non-CO_2_-corrected incubator at 37°C. Oxygen consumption rate (OCR) and extracellular acidification rate (ECAR) were measured using a Cell Mito Stress Kit (for OCR) kit in an XFp Analyzer (Seahorse Bioscience), with final concentrations of 1 μM oligomycin, 1 μM FCCP, and 0.5 μM rotenone/antimycin A. A detailed description of the assay can be found in [23]. Lactate was measured from cell culture supernatants using a coupled enzymatic assay in which lactate was oxidized and the resulting H_2_O_2_ was coupled to the conversion of Amplex Red reagent to fluorescent resorufin by HRP (horseradish peroxidase) [47].

### Ethics statement

Experimental work was conducted in agreement with the National Directive for Animal Protection (RD53/2013) for protection of experimental animals which meets the European Union Directive 2010/63 and with the approval from the Ethics Committees for Animal Experiments from the University of Zaragoza (PI46/18) and ISCIII (Proex 218/15). All animal procedures were performed by qualified researchers with the degree of “Personnel responsible for directing and design experimental animal procedures, Category C“, (Comunidad Autónoma de Aragón, Decreto 239/2008). Mice were maintained in the regulated Centro de Investigaciones Biomédicas de Aragón (CIBA, Zaragoza, Spain) facilities with reference number ES 50 297 0012 011. Buffy coats from healthy donors were obtained after written informed consent (Sanquin Blood Bank, Nijmegen, the Netherlands). Samples were anonymized to safeguard donor privacy.

### *In vivo* experimental models

For the *in vivo* trained immunity experiment, 8 weeks old female C57BL/6 JRJ (Janvier Biolabs) were vaccinated by the subcutaneous route with MTBVAC 10^6^ CFU and 4 weeks later, 10 μg/mice of LPS was administered by intraperitoneal injection. Mice were humanely sacrificed 4 hours later to collect the blood, by CO_2_ inhalation. All mice were anesthetized when receiving subcutaneous and intraperitoneal inoculations by inhalation with 5% of Isoflurane (Isoba Vet) using a vaporizer. The maintenance of the anaesthesia was performed at 1.5–2% of Isoflurane. Blood collection was carried out at the end point of the experiment, by cardiac puncture through the diaphragm using 25G needle, in order to collect 0.4–0.8 ml and the serum was obtained after centrifuging the blood for 10 min at 10xg.

For the pneumonia model of infection, female C57BL/6 mice bred at ISCIII animal facility were used. The *in vivo* trained immunity was performed in mice vaccinated by the subcutaneous route with MTBVAC 10^6^ CFU or PBS as placebo for the lethal control and 9 weeks later, *S*. *pneumoniae* infection was established. Briefly, mice under anaesthesia with 5% of isoflurane were inoculated by the intranasal route with 50 μl containing 1×10^4^ CFU/mouse of 957/13 strain of serotype 3. This is a *S*. *pneumoniae* clinical isolate from a 57 years old patient who suffered community-acquired bacteraemic pneumonia. Bacterial levels in blood, were determined from every infected mouse at 24 h, 48 h and 72 h post infection from a 6 μl sample of the tail vein. Mice were monitored for a period of 7 days. Experiments were repeated twice using 5 mice in each group and results were expressed as Log_10_ CFU/ml of bacteria recovered from the blood.

### Statistical analysis

Data are presented as mean ± SEM, as indicated in the legend of each figure, unless otherwise stated. The significance of the differences between groups was evaluated using Wilcoxon signed-rank test or Mann-Whitney test, as shown in the Figure Legends. Data are judged to be statistically significant when p < 0.05 by two-tailed Student’s t test. Survival was analyzed by the Log-rank (Mantel-Cox) test. For statistical analysis, data are compared with the control condition unless otherwise stated. In figs, asterisks denote statistical significance (*, p<0.05; **, p<0.01; ***, p<0.001).

## Supporting information

S1 Fig Effects of different strains on cytokine production. (A) IL-1β, IL-6, TNFα and IL-10 production by human monocytes 24 h after stimulation with different concentrations of MTBVAC, LPS or β-glucan in a medium without antibiotic (gentamicin). *p<0.05, Wilcoxon signed-rank test, compared to the control group. (B) IL-1β, IL-6, TNFα and IL-10 production by human monocytes 24 h after stimulation with different concentrations of BCG Pasteur, LPS or β-glucan in a medium with or without antibiotic (gentamicin). Mean ± SEM, n = 6; pooled from 2 independent experiments with 3 individual donors each. *p<0.05, Wilcoxon signed-rank test. w/o Ab: without antibiotic; ns: not significant; ctrl: control group.
(TIF)

S2 Fig MTBVAC stimulates cytokine production by human PBMCs. IL-1β, IL-6, TNFα, IL-10, IFNγ, IL-17 and IL-22 production by human monocytes 48 h or 7 days after a single stimulation of PBMCs with different doses of MTBVAC. Mean ± SEM, n = 3.
(TIF)

S3 Fig Comparison of MTBVAC and BCG Pasteur as stimuli for the induction of trained immunity. IL-6 and TNFα produced by human monocytes stimulated with MTBVAC, BCG Pasteur LPS or β-glucan, with or without antibiotic (gentamicin) in the medium, for 24 h and restimulated with LPS 6 days later. Mean ± SEM, n = 6–9; pooled from 2–3 independent experiments with 3 individual donors each. *p<0.05, Wilcoxon signed-rank test, compared to the control group. (w/o Ab: without antibiotic, ctrl: control)
(TIF)

S4 Fig Lactate production induced by different concentrations of BCG in human monocytes. (A) Lactate production by human monocytes 6 days after 24h-stimulation with different concentrations of BCG Pasteur, with or without antibiotic (gentamicin). (n = 6–9; pooled from 2–3 independent experiments) *p<0.05, Wilcoxon signed-rank test, compared to the control group unless otherwise stated. (B) Basal and maximum (Max) oxygen consumption rate (OCR) and extracellular acidification rate (ECAR) of monocytes were determined 6 days after 24h-stimulation with BCG or MTBVAC by extracellular flux measurements (mean ± SEM, n = 3). w/o Ab: without antibiotic; ctrl: control.
(TIF)

S5 Fig MTBVAC stimulates ROS production by trained monocytes. ROS production of human monocytes treated with MTBVAC and BCG Pasteur with or without 6-aminonicotinamide (6-AN). (n = 6; pooled from 2 independent experiments). *p<0.05, Wilcoxon signed-rank test, compared to the control group. ns: not significant; ctrl: control.
(TIF)

S6 Fig Epigenetic effects of MTBVAC, BCG and β-glucan. (A) H3K4me3 marks were assessed at the level of promoters of *TNFΑ* and *IL6* with two different pairs of primers after stimulation with BCG Pasteur, MTBVAC or RPMI (ctrl) (n = 6; pooled from 2 independent experiments). *p<0.05, Wilcoxon signed-rank test, compared to the control group. (B) H3K4me3 marks at the level of promoters of *TNFΑ* and *IL6* with two different pairs of primers after stimulation with MTBVAC, β-glucan or RPMI (control); mean ± SEM, n = 3.
(TIF)
