# Supplementary figures and images for: New live attenuated tuberculosis vaccine MTBVAC induces trained immunity and confers protection against experimental lethal pneumonia
Source: PLoS Pathog. 2020 Apr 2;16(4):e1008404. doi: 10.1371/journal.ppat.1008404 (PMC7117655; doi:10.1371/journal.ppat.1008404)

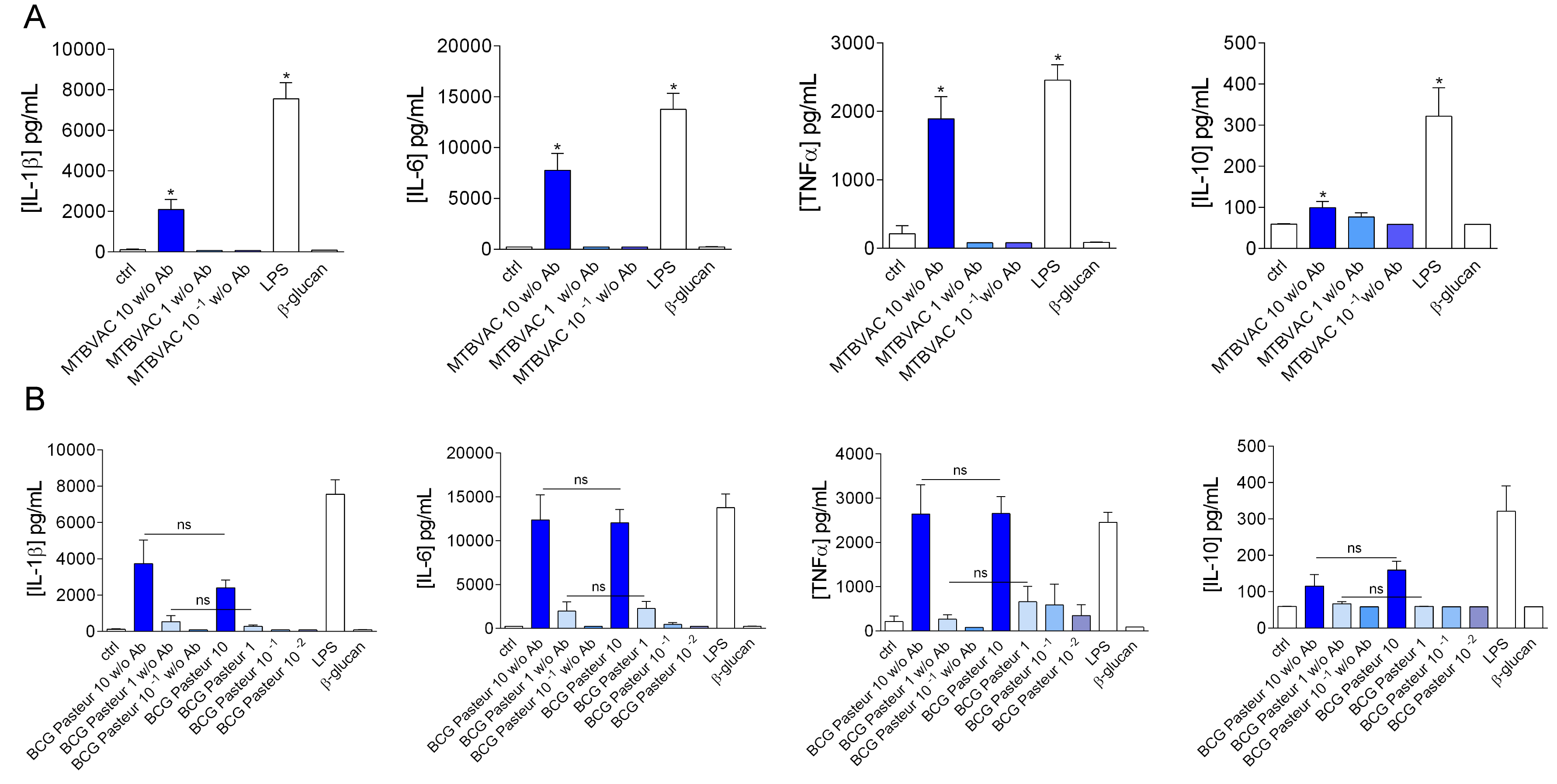

Supplement: S1 Fig — (A) IL-1β, IL-6, TNFα and IL-10 production by human monocytes 24 h after stimulation with different concentrations of MTBVAC, LPS or β-glucan in a medium without antibiotic (gentamicin). *p<0.05, Wilcoxon signed-rank test, compared to the control group. (B) IL-1β, IL-6, TNFα and IL-10 production by human monocytes 24 h after stimulation with different concentrations of BCG Pasteur, LPS or β-glucan in a medium with or without antibiotic (gentamicin). Mean ± SEM, n = 6; pooled from 2 independent experiments with 3 individual donors each. *p<0.05, Wilcoxon signed-rank test. w/o Ab: without antibiotic; ns: not significant; ctrl: control group. (TIF) [file ppat.1008404.s001.tif]

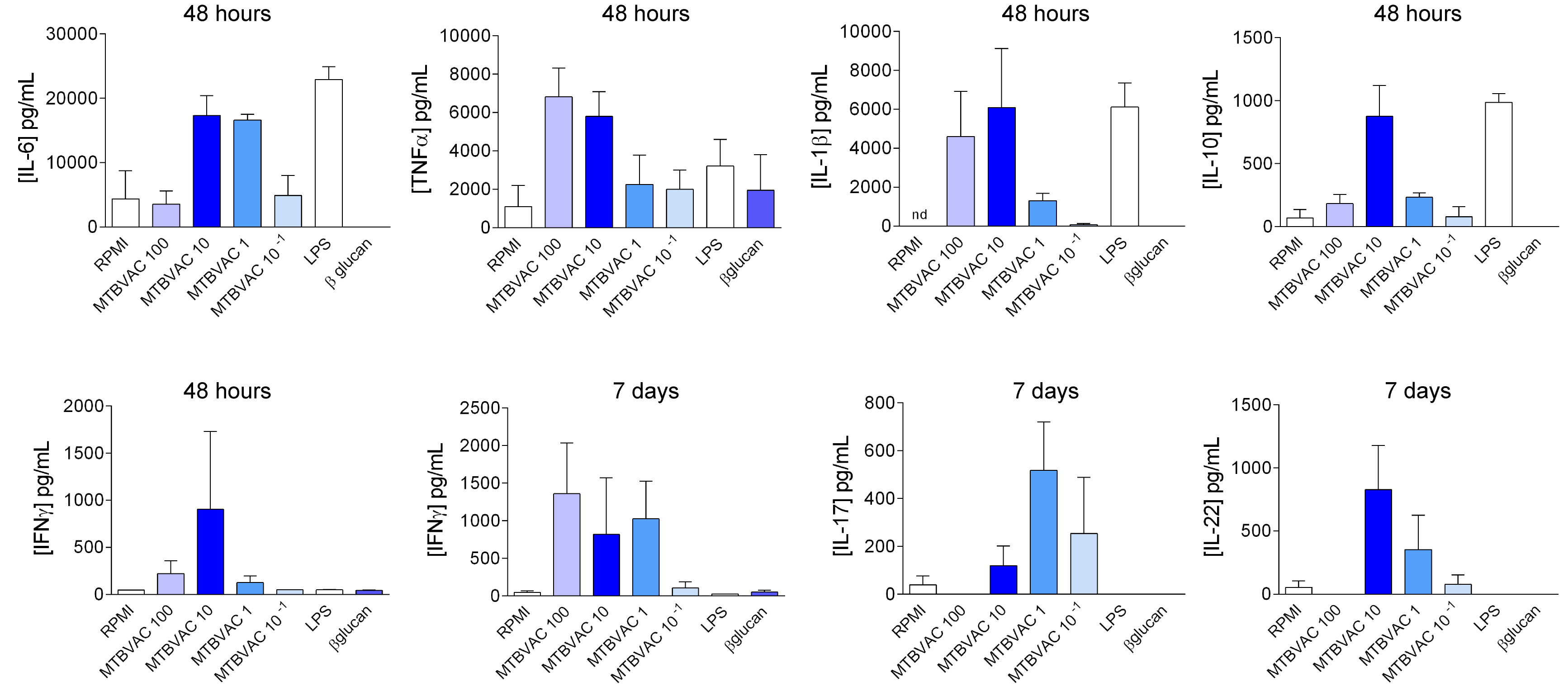

Supplement: S2 Fig — IL-1β, IL-6, TNFα, IL-10, IFNγ, IL-17 and IL-22 production by human monocytes 48 h or 7 days after a single stimulation of PBMCs with different doses of MTBVAC. Mean ± SEM, n = 3. (TIF) [file ppat.1008404.s002.tif]

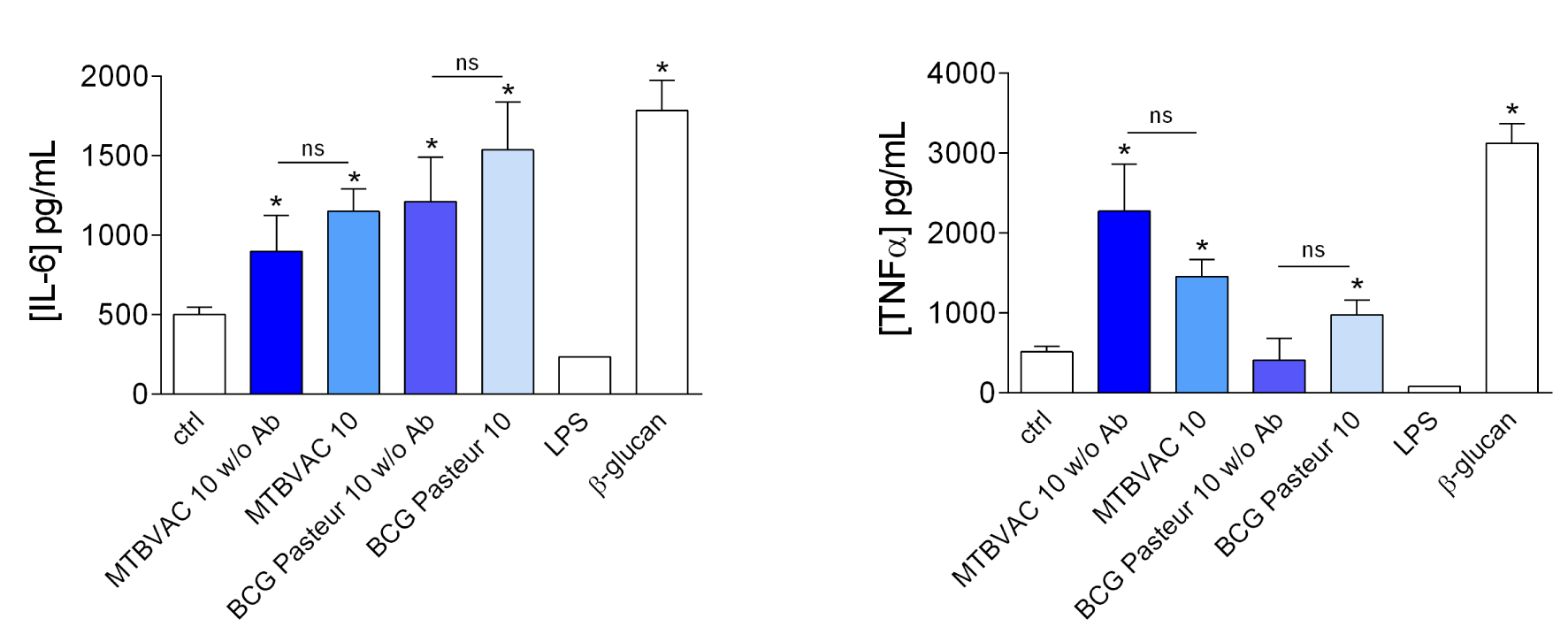

Supplement: S3 Fig — IL-6 and TNFα produced by human monocytes stimulated with MTBVAC, BCG Pasteur LPS or β-glucan, with or without antibiotic (gentamicin) in the medium, for 24 h and restimulated with LPS 6 days later. Mean ± SEM, n = 6–9; pooled from 2–3 independent experiments with 3 individual donors each. *p<0.05, Wilcoxon signed-rank test, compared to the control group. (w/o Ab: without antibiotic, ctrl: control) (TIF) [file ppat.1008404.s003.tif]

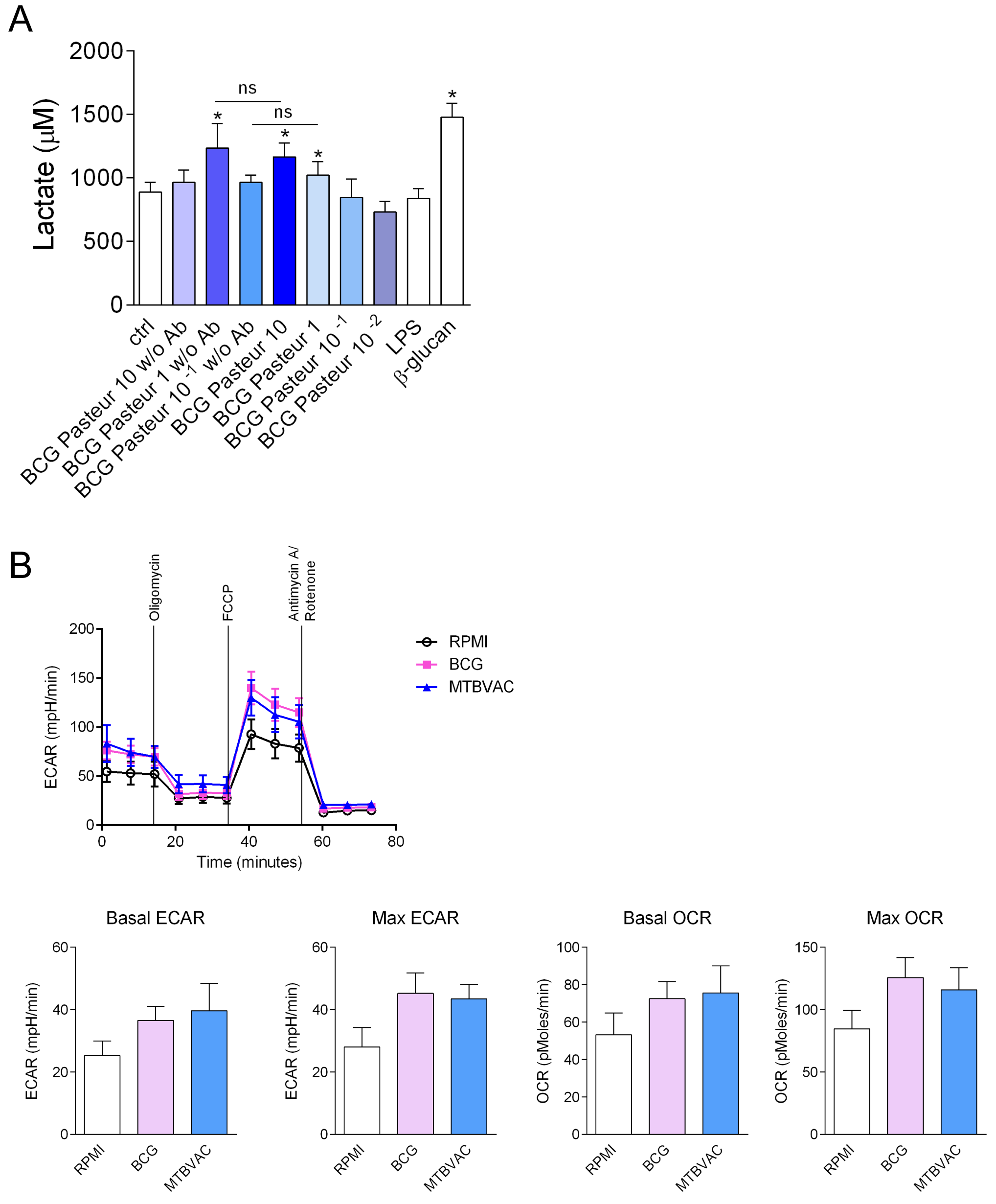

Supplement: S4 Fig — (A) Lactate production by human monocytes 6 days after 24h-stimulation with different concentrations of BCG Pasteur, with or without antibiotic (gentamicin). (n = 6–9; pooled from 2–3 independent experiments) *p<0.05, Wilcoxon signed-rank test, compared to the control group unless otherwise stated. (B) Basal and maximum (Max) oxygen consumption rate (OCR) and extracellular acidification rate (ECAR) of monocytes were determined 6 days after 24h-stimulation with BCG or MTBVAC by extracellular flux measurements (mean ± SEM, n = 3). w/o Ab: without antibiotic; ctrl: control. (TIF) [file ppat.1008404.s004.tif]

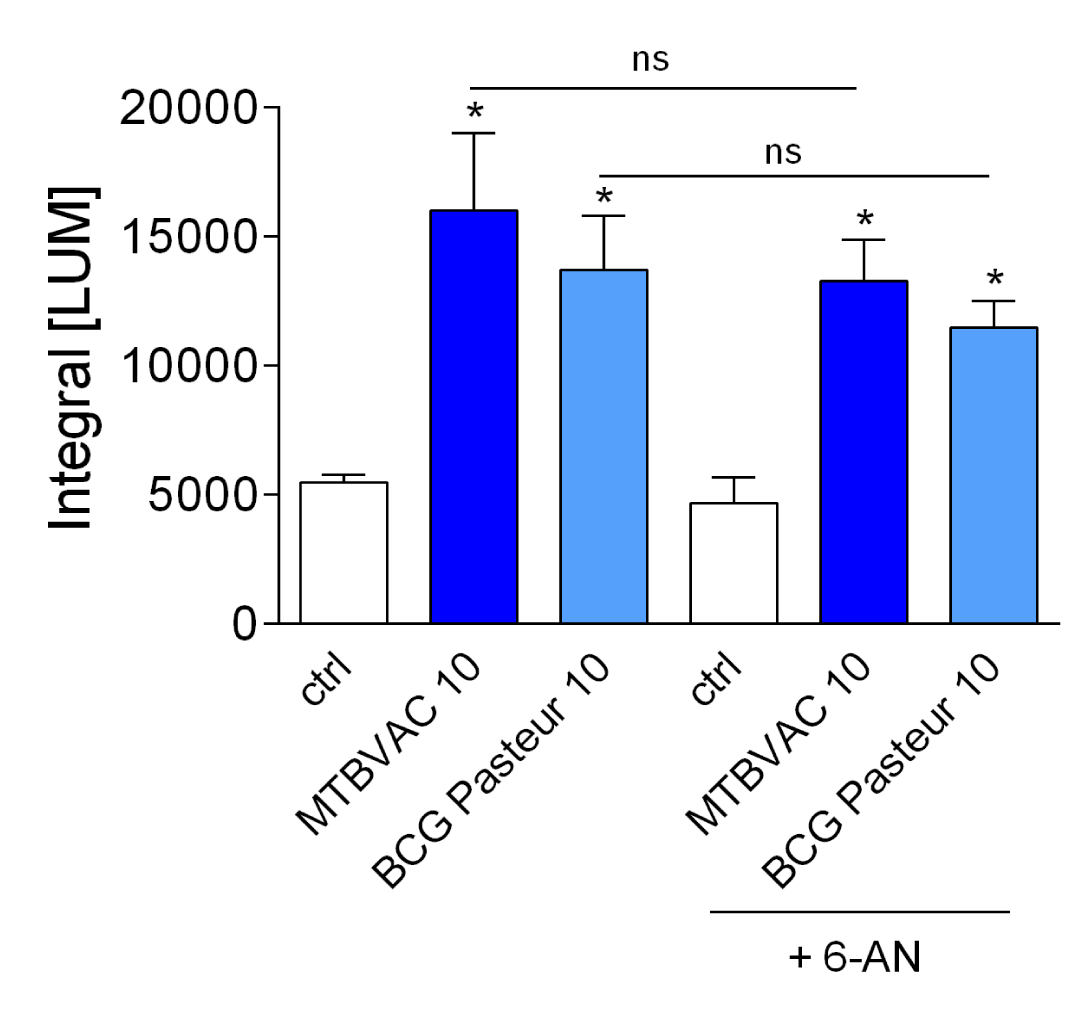

Supplement: S5 Fig — ROS production of human monocytes treated with MTBVAC and BCG Pasteur with or without 6-aminonicotinamide (6-AN). (n = 6; pooled from 2 independent experiments). *p<0.05, Wilcoxon signed-rank test, compared to the control group. ns: not significant; ctrl: control. (TIF) [file ppat.1008404.s005.tif]

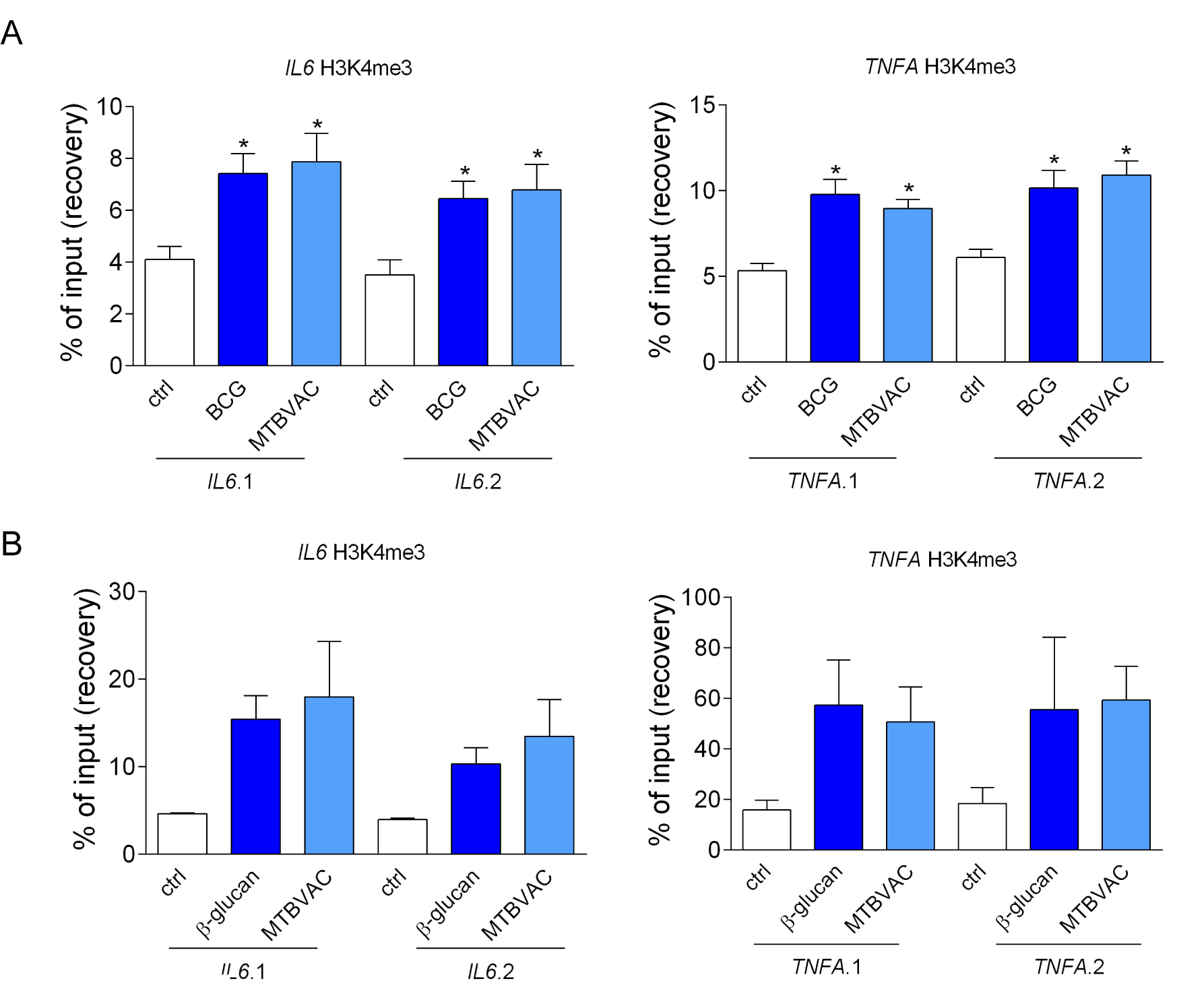

Supplement: S6 Fig — (A) H3K4me3 marks were assessed at the level of promoters of TNFΑ and IL6 with two different pairs of primers after stimulation with BCG Pasteur, MTBVAC or RPMI (ctrl) (n = 6; pooled from 2 independent experiments). *p<0.05, Wilcoxon signed-rank test, compared to the control group. (B) H3K4me3 marks at the level of promoters of TNFΑ and IL6 with two different pairs of primers after stimulation with MTBVAC, β-glucan or RPMI (control); mean ± SEM, n = 3. (TIF) [file ppat.1008404.s006.tif]
